# Structural gender inequality and gender differences in adolescent substance use: A multilevel study from 45 countries

**DOI:** 10.1016/j.ssmph.2022.101208

**Published:** 2022-09-06

**Authors:** Alina Cosma, Frank J. Elgar, Margreet de Looze, Natale Canale, Michela Lenzi, Jo Inchley, Alessio Vieno

**Affiliations:** a Department of Sociology, Trinity College Dublin, Dublin, Ireland; b Department of Developmental and Social Psychology, University of Padova, Padova, Italy; c Sts Cyril and Methodius Faculty of Theology, Olomouc University Social Health Institute, Palacky University in Olomouc, Olomouc, Czech Republic; d School of Population and Global Health, McGill University, Montreal, Canada; e Department of Interdisciplinary Social Science, Utrecht University, Utrecht, the Netherlands; f MRC/CSO Social and Public Health Sciences Unit, University of Glasgow, Glasgow, United Kingdom

**Keywords:** Alcohol use, Tobacco use, Drunkenness, Gender, Health inequalities, Health Behaviour in School-aged Children study

## Abstract

- Societal gender inequality relates to gender differences in adolescent substance use.
- The gender gap in adolescent substance use is larger in countries with higher levels of gender inequality.
- Girls in these countries were less likely to get drunk, use alcohol or smoke cigarettes than boys.

## 1 Introduction

Substance use is a global public health concern that affects different populations, including adolescents (Schulte & Hser, 2013). Alcohol and tobacco use, in particular, are major contributors to global disease burden and direct economic costs to society (Degenhardt et al., 2018). Initiation of these behaviours typically occurs in adolescence (Degenhardt et al., 2018) when it can interfere with brain development (e.g., synaptic growth and pruning, especially in the prefrontal cortex), physiological changes (e.g., puberty), and psychosocial and educational (e.g., role transitions) challenges. As such, the initiation of substance use during the adolescent years places young people to immediate and long term health risks. Previous literature regarded this as a “triple risk” situation, involving immediate social and health effects, impacts on social transitions into adulthood, and effects on the offspring of young adults (Hall et al., 2016). Although adolescent substance use has declined markedly since the beginning of the 21st century (e.g., De Looze et al., 2019a), young people’s tobacco and alcohol use levels are still among the highest in Europe and North America compared to other regions (Degenhardt et al., 2018; ESPAD Group, 2020; Inchley et al., 2020).

Historically, boys reported higher levels of substance use than girls. In recent decades however, gender convergence in adolescent substance use has been observed in some, mostly Western, countries (Kuntsche et al., 2011; Vieno et al., 2013). Moreover, in some countries the traditional patterns of males engaging more in excess drinking and smoking than females has reversed (e.g., Austria, Sweden, Latvia etc), with young adolescent girls reporting higher prevalence than males (ESPAD Group, 2020; Hibell et al., 2012). Understanding the potential mechanisms for these cross-national variations in gender differences in adolescent substance use supports the United Nations Sustainable Development Goals 2030 (UN SDG 2030) agenda that aims to reduce gender inequalities in health. Based on the assumption that societal gender inequality reflects the extent to which boys and girls are expected to conform with traditional gender norms (i.e., boys should be active and risk-taking, whereas girls should be passive and well behaved) (Courtenay, 2000), this study examines whether gender differences in adolescent substance use relate to societal gender inequality.

### 1.1 Gender differences in substance us

Presently, substance use is still higher among boys than girls in most European and North American countries, however wide cross-national variations exist. For example, the most recent survey cycle of the European School Survey Project on Alcohol and Other Drugs (ESPAD) study (ESPAD Group, 2020) showed that in 2019, boys were more likely to report lifetime alcohol use than girls across Europe, with the largest gender difference being observed in Kosovo (41% for boys versus 18% for girls). In 16 out of 35 countries, however, the rate for girls was higher than that for boys, particularly in Lithuania (83% for girls versus 75% for boys) and Ukraine (89% versus 81%). Regarding alcohol intoxication (i.e., drunkenness), slightly more boys (14%) than girls (13%) reported that they had been intoxicated in the last 30 days, with the highest differences found in Serbia (15% for boys versus 10% for girls) and Montenegro (10% versus 5%). In Spain, more girls than boys reported alcohol intoxication in the last 30 days (19% for girls versus 14% for boys). While gender differences in tobacco use were small overall, in some countries (such as Kosovo, Georgia and Ukraine) boys were more likely to smoke, while in other countries (e.g., Bulgaria, Slovakia, and Spain) rates were higher among girls than boys. Such cross-national variability in gender differences in adolescent substance use suggests that contextual factors might establish or reinforce the gendered nature of substance use during adolescence.

### 1.2 Gender (in)equality and adolescent substance use

Societal gender inequality, measured as men’s and women’s unequal share of paid work, educational level, health, and political decision-making power in society as per the Gender Inequality Index (GII), varies considerably across Europe (UNDP, 2021). Research indicates there is a link between societal gender inequality and gender differences in a range of adolescent health behaviours and outcomes. For instance, gender differences in fighting and physical activity are higher in more gender unequal countries, due to the fact that boys in these countries are more likely to engage in these behaviours (de Looze et al., 2019b). Gender differences in injuries were also larger in gender unequal countries, but this was due to the fact that girls in these countries were less likely to report injuries, as compared to girls in more gender equal countries (de Looze et al., 2019b). Furthermore, greater gender inequality at country level was associated with larger gender differences in traditional bullying (perpetration and victimization). In contrast, lower gender inequality was associated with larger gender differences for cyber victimization (Cosma et al., 2022).

Societal gender inequality may influence boys' and girls' health behaviours and outcomes through prevailing traditional gender norms within countries and through societal restrictions on boys' and girls' behaviour (Slabbert et al., 2020). From a social constructionist perspective, adolescents act in accordance with normative expectations that reinforce gender roles (Kimmel, 2000). In countries where traditional gender norms prevail, adolescent boys may be more likely to exhibit behaviours that are considered ‘masculine’ (according to these norms) such as substance use, whereas girls in these countries are less likely to exhibit these behaviours (Slabbert et al., 2020). At the same time, girls in these countries may encounter more practical barriers to engaging in specific behaviours, ranging from membership in sports clubs and getting access to substances to experiencing difficulties regarding their position in the labour market, their reproductive health, and possibilities for empowerment (Heise et al., 2019).

Qualitative studies have also explored the role of social and gender norms in adolescent substance use. However, the caveat is that most of these studies were undertaken in mostly Western countries. For example, the association between so-called ‘masculine’ behaviour and heightened levels of health risk behaviours in adolescent girls may be connected to trying to “fit in” with peer groups engaging in risky behaviours (Lyons & Willott, 2008). Furthermore, while young women view alcohol drinking as a pleasurable aspect of their social lives, they report challenges in engaging in a traditionally ‘masculine’ behaviour whilst maintaining a desirable ‘femininity’. As such, they report having to manage a “balancing act” between femininity, retaining control and respectability whilst drinking (Lennox et al., 2018). This might point to the fact that women and adolescent girls in Western societies still face a gendered double standard when engaging in substance use. Furthermore, previous longitudinal data confirm that gender, gender roles, and social norms act both directly and interactively to predict substance use during adolescence (Mahalik et al., 2015). In particular, it seems that male typicality of a behaviour is relevant for girls' involvement in health risk behaviours (Mahalik et al., 2015). These findings support previous empirical research that found female’s adoption of masculine gender roles to have negative health effects.

### 1.3 Theoretical considerations

*Gender* is constructed from cultural and subjective meanings about what constitutes typical masculine or feminine behaviour. It constantly shifts and varies, depending on cultural and societal norms and expectations (Courtenay, 2000; Kimmel, 2000). From *social constructivist and feminist perspectives*, the display of health behaviours are a means for constructing and demonstrating femininities and masculinities (Courtenay, 2000), and these are always culturally defined. Indeed, young men who endorse dominant norms of masculinity engage in more risk-taking behaviours, including higher levels of substance use (Courtney, 2000). However, more recently, a softened version of masculinity seems to be endorsed by boys and young men, especially in Western countries (Roberts, 2013) which could be due to the transition in most of the countries to a more gender equal society and culture. However, it is important to note that the ‘appropriate’, respected and even hegemonic way of ‘being a man’ is contingent upon the social, historical and, often, the very specific context (Roberts, 2013).

Another mechanism by which societal gender inequality may affect gender differences in adolescent behaviour is *exposure opportunity –* the notion that males have more opportunities to use substances (Wells et al., 2011). Previous research found that opportunities to use alcohol are widespread in high income countries in Europe (Wells et al., 2011) but more limited in Eastern Europe. At the same time, as part of a cultural change in which women are encouraged to define themselves by balancing the traditional feminine characteristics and embracing self-entitlement, more empowered and confident notions of femininity have emerged in many countries in the last decades (Budgeon, 2014; Ringrose, 2007). Therefore, we would expect adolescent girls’ substance use to be particularly sensitive to changes in gender inequality at country level.

### 1.4 Current study

This paper builds on previous work that investigated the role of structural gender inequality and adolescent health behaviours (Cosma et al., 2022; de Looze et al., 2019a, De Looze et al., 2019b; de Looze et al., 2018). On one hand, structural gender inequality seems to be positively associated with adolescents’ life satisfaction with adolescents in countries with relatively higher levels of gender equality reporting higher life satisfaction (de Looze et al., 2018). On the other hand, structural gender inequality has a differential effect on males and females, thus altering gender differences in behaviours such as fighting, injuries, bullying or cyberbullying is associated with gender differences in behaviours such as fighting, injuries, bullying or cyberbullying (Cosma et al., 2022; de Looze et al., 2019a, De Looze et al., 2019b). The unique contribution of this study is the examination of gender differences in adolescent substance use in relation to societal gender inequality. Its aims were to document gender differences in adolescent substance use (i.e., alcohol drinking and tobacco smoking) across 45 countries and regions and then examine whether national level gender inequality relates to gender differences in adolescent substance use. Based on a feminist theoretical perspective which considers gender differences to be related to societal power and control imbalance that exists between men and women, we hypothesize that gender differences in adolescent substance use is positively related to national-level gender inequality (i.e., greater gender inequality will be accompanied by larger gender differences). Given that previous research argues that men and boys experience greater social pressure than women and girls to endorse gendered societal prescriptions (Courtenay, 2000), we expect that the gender gap in adolescent substance use is positively correlated with gender inequality.

## 2 Methods

### 2.1 Sample

The data used in this study were collected in Health Behaviour in School-aged Children (HBSC) study, a large cross-sectional, school-based survey carried out every four years in collaboration with the WHO Regional Office for Europe. We used data from the 2017/2018 HBSC survey, in which 47 countries or regions participated by collecting self-report data on nationally representative samples of 11-, 13-, and 15-year-old adolescents using a standardized study protocol (Inchley et al., 2018). Samples were drawn using cluster sampling, with school classes or the whole school as the primary sampling unit. Data collection procedures and questionnaires were standardized and strictly followed the international research protocol, including standardized translation and backtranslation of their national questionnaires. Each participating country obtained ethical board approval from national level accredited organisation. The present study includes data from 45 countries and regions that included items on tobacco use, alcohol use and drunkenness. The median response rate at the school level was 82.7% (interquartile range, 48.6%–92.8%) and at the individual level 83.0% (interquartile range, 70.7%–87.5%).

### 2.2 Individual level variables

Substance use (cigarettes smoking, alcohol drinking, drunkenness). Adolescents were asked the frequency in days in which they 1) smoked cigarettes, 2) drank alcohol, and 3) had been drunk in *i)* the last 30 days, and *ii*) their lifetime. For each question, the response options ranged from “zero” to “30 days or more”, respectively for the drunkenness items ranged from (1) “no, never” to (5) “yes, more than 10 times”. These questions have been widely used and validated for cross-national use (Horváth et al., 2021; Inchley et al., 2018). For the purpose of this paper, all responses were recoded into a binary variable so that adolescents who indicated they used any of the substance at least once or more were coded as ‘1’ and those who reported that they did not use these substances where assigned the reference group (‘0’).

Gender: Adolescents were asked to indicate whether they are a boy or a girl. In the subsequent analysis, the reference category was set to those reported they are a girl.

Individual level control variables. At the individual level, control variables included in the regression models were *age* category (11-, 13- and 15-year-olds) and *material deprivation*. The latter was based on the HBSC Family Affluence Scale (FAS), a 6-item measure of material assets in the household (e.g., cars, computers) (Torsheim et al., 2016). These data were transformed to a weighted proportional rank index of material deprivation within each country that ranged from 0 (least deprivation, most affluent) to 1 (most deprivation, least affluent).

### 2.3 Country level variables

Gender inequality. We used the Gender Inequality Index (GII) from year 2018 of the United Nations Development Program (UNDP) (United Nations Development Program (UNDP) (2021) as a measure of human development costs of gender inequality. This composite index combines a reproductive health dimension (maternal mortality ratio and adolescent fertility rate), an empowerment dimension (proportion of parliamentary seats occupied by females, and proportion of adult females and males aged 25 years and older with at least some secondary education) and a labour dimension (expressed as labour market participation and measured by labour force participation rate of female and male populations aged 15 years and older). The GII takes values from 0 to 1, with higher values indicating greater levels of gender inequality within each given society. An overview of the GII values for the countries included in this study is presented in Supplemental material (Table S1).

Gross domestic product (GDP)*per capita*. To control for differences in country wealth we used data on national GDP per capita, PPP (current international $) in the data bank of the World Bank converted to thousands of dollars (World Bank, 2021).

### 2.4 Analytical approach

Stata/SE v. 16 (College Station, TX) was used for statistical analysis. The 2017/18 HBSC data were linked to country-level data on gender inequality (GII) and country wealth (GDP per capita). In the first step, we calculated the weighted prevalence of each of the substance use outcomes in males and females, with adjustment for socioeconomic differences and sample weights. For each of the investigated outcomes, forest plots of odds ratios obtained from regressions were created using the *coefplot* command. The main analyses consisted of separate multilevel mixed effects logistic regression analyses of each dependent variable: alcohol drinking (lifetime and last 30 days); tobacco smoking (lifetime and last 30 days) and drunkenness (lifetime and last 30 days). Values of GII and GDP per capita were included at the country level and gender, age group family deprivation and the interaction of gender and GII were included as individual-level variables. The *margins* and *marginplot* commands were used to display interactions between gender and country-level gender inequality. Mixed effects logistic regression models were fitted using the Stata command *melogit*. The analyses allowed for random effects of gender inequality at the country level and to cluster in the data at school and national levels. As such, the models specified three levels of random variation among countries (n = 45), schools (n = 8248) and individual students (n = 224,876) and were weighted to ensure the results represented national populations of 11-, 13-, and 15-year-olds. A significant positive interaction of male gender and national gender inequality was interpreted as supportive of our hypothesis. Stratified analyses at the country level were also weighted and adjusted for the clustered sample design using Stata’s *svy* command.

*Sensitivity analysis.* We also ran fixed effects regression models to test these interactions. This more conservative approach adjusts for any unmeasured country differences that are constant over time but vary across individuals by explicitly including a separate intercept term for each country (αi) as dummy variables in the regression equation. Furthermore, Azerbaijan only asked the substance use items to the 15-year-olds and partly to 13-year-olds in their sample and, therefore, had on average 46% missing data. To ensure our results were not biased by this, we tested the multilevel mixed-effects model with and without data from Azerbaijan.

## 3 Results

### 3.1 Cross-national variation in gender gaps in substance use

Table 1 presents descriptive statistics on the key variables included in the study. Overall, the most prevalent form of substance use was the lifetime use of alcohol (34.63%), whereas the lowest prevalence was observed in drunkenness in the past 30 days (6.33%) and cigarette smoking in the past 30 days (6.57%). The rates of alcohol use, tobacco smoking and drunkenness (lifetime and last 30 days) by gender are illustrated for each country in Supplementary Tables S4–S9, respectively. Across all substances in most countries there were no significant gender differences. However, in countries where we identified significant gender differences, it was mostly boys that reported higher rates of use than girls. The largest absolute percentage point gender differences were observed for lifetime alcohol use (17.39% higher in boys in Israel) and lifetime drunkenness (15.34% higher in boys in Georgia). A small number of countries showed higher rates of substance use in girls (e.g., 4.17% more lifetime smoking in girls in Italy).

**Table 1:** Descriptive statistics on key variables.

	n	Unweighted %	Weighted %
*Individual level* (*n =* 224,876)			
Gender group			
Male	110,168	48.99	49.18
Female	114,708	51.01	50.82
Missing	0.00	0.00	0.00
Age group			
11 years	74,442	33.10	33.27
13 years	77,511	34.47	34.04
15 years	72,539	32.26	32.52
Missing	384	0.17	0.17
Smoking – lifetime			
Once or more	29,721	13.22	13.19
Never	183,117	81.43	81.48
Missing	12,038	5.35	5.33
Smoking – past 30 days			
Once or more	14,758	6.56	6.57
Never	198,549	88.29	88.31
Missing	11,569	5.14	5.13
Alcohol use - lifetime			
Once or more	77,492	34.46	34.63
Never	135,927	60.45	60.24
Missing	11,457	5.09	5.13
Alcohol use – past 30 days			
Once or more	41,124	18.29	18.42
Never	172,692	76.79	76.61
Missing	11,060	4.92	4.97
Drunkenness – lifetime			
Once or more	34,099	15.16	15.32
Never	179,084	79.64	79.47
Missing	11,693	5.20	5.21
Drunkenness – past 30 days			
Once or more	14,053	6.25	6.33
Never	198,718	88.37	88.29
Missing	12,105	5.38	6.33

	Mean	Std. deviation	missing (%)
Age (years)	13.51	1.63	1.93
Material deprivation	0.50	0.29	4.30

*Country level (n = 45)*			
GDP per capita, $000s	41.65	21.02	0.00
Gender inequality	0.13	0.08	0.00

These gender differences across countries are also displayed in a series of forest plots in Supplementary Figs. S1–S3. The supplement also shows scatterplot charts of country-level prevalence estimates in males versus females (Fig. S4) and correlations of gender differences in substance use and country gender inequality (Fig. S5).

### 3.2 Structural gender inequality and substance use

The results of the multilevel logistic regressions of adolescent substance use are shown in Table 2. The interaction of country-level gender inequality and gender was associated with all six forms of substance use.

**Table 2:** Multilevel regression of substance use in adolescents including interactions of gender and gender inequality (HBSC 2017/18).

	Smoking (lifetime)		Smoking (past 30 days)		Alcohol use (lifetime)		Alcohol use (past 30 days)		Drunkenness (lifetime)		Drunkenness (past 30 days)	
	OR	95% CI	OR	95% CI	OR	95% CI	OR	95% CI	OR	95% CI	OR	95% CI
Gender (male)	1.00	(0.95–1.06)	0.88***	(0.82–0.95)	1.24***	(1.19–1.29)	1.00	(0.95–1.05)	0.99	(0.94–1.04)	0.95	(0.88–1.02)
Age group												
11y	1	(1.00–1.00)	1.00	(1.00–1.00)	1.00	(1.00–1.00)	1.00	(1.00–1.00)	1.00	(1.00–1.00)	1.00	(1.00–1.00)
13y	3.82***	(3.63–4.02)	3.43***	(3.18–3.69)	3.22***	(3.12–3.32)	3.45***	(3.30–3.60)	3.15***	(3.01–3.31)	2.95***	(2.73–3.18)
15y	12.50***	(11.91–13.13)	12.85***	(11.98–13.79)	11.54***	(11.16–11.93)	13.20***	(12.65–13.78)	14.21***	(13.57–14.88)	13.26***	(12.35–14.25)
Gender inequality (GII)	0.05	(0.00–1.04)	0.12	(0.01–1.45)	0.02*	(0.00–0.91)	0.03	(0.00–1.06)	0.34	(0.01–8.81)	0.18	(0.01–2.54)
Deprivation	1.00	(0.95–1.05)	1.08*	(1.01–1.15)	0.65***	(0.63–0.68)	0.60***	(0.57–0.63)	0.72***	(0.69–0.76)	0.69***	(0.65–0.74)
Country wealth (GDP pc)	0.99	(0.98–1.00)	0.99	(0.98–1.00)	0.99	(0.98–1.01)	0.99	(0.98–1.00)	0.99	(0.98–1.00)	0.99	(0.98–1.00)
Gender * GII	4.57***	(3.08–6.78)	6.07***	(3.63–10.14)	1.95***	(1.45–2.63)	5.06***	(3.57–7.16)	6.45***	(4.49–9.27)	9.81***	(5.87–16.41)

*Simple effects:*												
*GII (in females)*	*0.05*	*(0.00, 2.14)*	0.15	*(0.00, 4.61)*	*0.01*	*(0.00, 1.06)*	*0.03*	*(0.00, 1.51)*	*0.52*	*(0.01, 18.34)*	*0.29*	*(0.01, 6.15)*
*GII (in males)*	0.18*	*(0.01, 2.93)*	0.46	*(0.05, 3.91)*	*0.04*	*(0.00, 1.77*	*0.14*	*(0.00, 4.69)*	*1.59*	*(0.07, 36.99)*	1.11*	*(0.09, 14.17)*

Constant	0.05***	(0.02–0.11)	0.02***	(0.01–0.04)	0.30*	(0.11–0.85)	0.10***	(0.04–0.27)	0.05***	(0.02–0.13)	0.02***	(0.01–0.04)

Variances:												
Country	0.34		0.22		0.56		0.49		0.39		0.25	
School	0.29		0.36		0.33		0.34		0.30		0.38	
ICC (country)	0.03		0.02		0.05		0.03		0.03		0.01	
ICC (school)	0.13		0.09		0.20		0.16		0.14		0.08	
VIF	3.99		3.98		4.02		4.01		4.02		4.02	
*N*	212469		212938		213053		213445		212809		212406	

Note: OR = odds ratio. CI = confidence interval. ICC = intraclass correlations. VIF = mean variance inflation factor. Simple effects of the interaction, shown in italics, were tested in separate models.

*p < 0.05; **p < 0.01; ***p < 0.001.

**Fig. 1:**
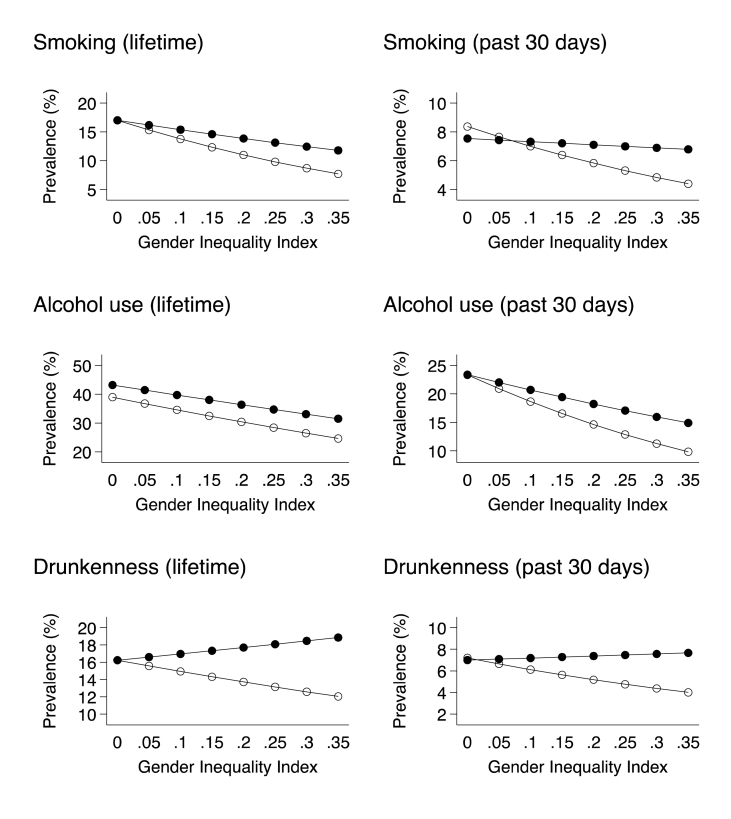
Prevalence of substance use in boys (black circles) and girls (white circles) by country gender inequality. Estimates are weighted and adjusted for differences in age group, material deprivation and country wealth.

The direction of these interactions is displayed in the marginal effects of gender inequality for males and females in Fig. 1. In each case, gender differences in substance use were greater at higher gender inequality due to a steeper decline in girls as compared to boys. In the case of smoking and alcohol use (lifetime and past 30 days), both gender groups showed less substance use at higher gender inequality, however the trend was stronger in females. A slightly different pattern was found in drunkenness (lifetime and past 30 days) where higher gender inequality related to lower prevalence in girls only and either no change or a slight increase in boys. Overall, girls in countries with higher levels of gender inequality are less likely than boys to smoke cigarettes, use alcohol and get drunk. Variance inflation factors (VIF) for all variables in all models did not exceed 6.33, suggesting the results were not biased by multicollinearity between variables (VIF <10; Kennedy, 2003). Mean VIF estimates across all six models ranged from 3.98 to 4.02.

### 3.3 Social patterns in substance use

With respect to the control variables, we observed large differences in substance use between age groups, with odds ratios ranging from 11.54 (95% CI 11.16–11.93) in alcohol use (lifetime) to 14.21 (95% CI 13.57–14.88) in drunkenness (lifetime) in 15-year-olds as compared to 11-year-olds. Material deprivation was *positively* associated with smoking in the past 30 days (odds ratio = 1.08 [95% CI 1.01–1.15]). However, deprivation was *negatively* associated with all four indicators of alcohol use and drunkenness, with odds ratios ranging from 0.72 (95% CI 0.69–0.76) to 0.60 (95% CI 0.57–0.63). With all other variables considered, country wealth did not relate to any form of substance use.

### 3.4 Sensitivity analyses

To ensure these results were not biased by unmeasured country-level variables, we repeated the analyses using fixed effects regression (Table S3). The results again showed strongly significant associations of the interaction of gender and gender inequality with all six measures of substance use, thus confirming the robustness of our findings. We checked the robustness of the findings with Azerbaijan removed and found no difference in the overall pattern of interactions between gender and GIIs (Table S2).

## 4 Discussion

While adolescent tobacco and alcohol use were typically more prevalent among boys than girls up until the beginning of the 21st century, large variations now exist in the direction and magnitude of these gender differences. This study used data on 45 European and North American countries to examine whether these gender differences could be explained by national-level gender inequality. Our findings indicate that the gender gap in adolescent substance use was larger in countries with higher levels of gender inequality. Specifically, girls in these countries were less likely to get drunk, use alcohol or smoke cigarettes than boys.

One explanation for this pattern is that substance use is typically considered a risky - and thus, according to traditional gender roles, a more masculine - behaviour, which is less socially acceptable for girls, especially in countries with high levels of gender inequality. Previous research shows robust correlations between structural gender inequality and gender norms (as measured by Gender Social Norms Index) (Cosma et al., 2022) and both are recognised to be powerful but separate determinants of health and well-being (Heise et al., 2019). Furthermore, the gender gap in other externalizing behaviours such as school bullying, fighting or injuries seems to increase with increasing structural gender inequality with boys reporting higher involvement (Cosma et al., 2022; de Looze et al., 2019a, De Looze et al., 2019b). Biosocial theory (Wood & Eagly, 2002, 2012) posits that in countries characterised by higher gender inequality, parents differentiate more between their daughters and sons and socialisation processes within the family may reinforce more traditional gender roles in preparation for adulthood. Thus, in these countries, boys might have an inherently different and more autonomous role, leading them to be freer to experiment with drinking alcohol or smoking cigarettes than girls, while girls might be subjected to greater social control and different behavioural expectations (Hadjar et al., 2007). Furthermore, our findings lend support to the idea that exposure opportunity to substance use is higher among boys, but only in more gender unequal countries.

Besides the potential impact of restrictive gender norms, there are several other explanations for these findings. First, girls in more gender unequal countries may experience more practical barriers to substance use (Slabbert et al., 2020). Girls may have more difficulties purchasing substances or be less likely to find themselves in social situations (e.g., going out at night) that create opportunities to smoke or drink, for example, through stricter parental monitoring or lower parental acceptance of substance use. Girls in more gender equal countries, on the other hand, may enjoy more freedom and suffer less from the constraints of traditional gender roles, and may therefore be more likely to use substances (Cheng & Anthony, 2017). Second, the rise of girls consuming tobacco and alcohol could also be explained by the aggressive gendered marketing that links tobacco products and alcohol with female empowerment and other aspirations (Amos & Haglund, 2000; Atkinson et al., 2022). Similarly, gendered marketing techniques used by alcohol and tobacco companies, such as using fashion blogs in social media campaigns, promoting special events (e.g., offering free drinks for women), using recurrent themes for promoting their products in women (e.g., body image, weight control and gender equality) and developing specific flavours for women (e.g., menthol in cigarettes and fruit-flavoured beer) may encourage girls to adopt these behaviours (Thibaut, 2018).

While more behavioural freedom for girls in more gender equal countries has led to a range of positive outcomes, such as more equal access to the labour market, this study shows that greater equality, or emancipation, can also result in higher levels of engagement in harmful behaviours that form a threat to young women’s health. This may be considered a (negative) by-product of the (positive) trend towards more gender equality in many countries. Also, we can infer that greater structural gender equality towards the UN SDGs will result in positive changes in population health profiles, including adolescent health behaviour. However, as shown by our findings, especially for adolescent health, structural gender equality comes with mixed effects on individual behaviours. As societies progress towards more gender equality, a whole-system approach is needed to adapt to these changes (Backhans et al., 2007). Overall, the current study may be indicative of future developments in countries that currently score high on the Gender Inequality Index, but that are moving towards more gender equality. Looking to the future, these results may thus contribute to predicting changes in boys' and girls' health and health behaviours in countries going through social transitions towards more gender equality. Future studies should monitor these probable evolutions, especially areas such as substance use.

Increasing gender parity over time in substance-use rates, including disorders, are reported across the globe (Slade et al., 2016), and this could be attributed to declining traditional gender roles, especially in Western countries (Seedat et al., 2009). While, initially, this (convergence in substance use) was due to increase prevalence among girls, there has been a decline among both girls and boys in recent years – perhaps due to successful health promotion policies and programmes, as well as secular changes in adolescent time use. In addition, recent evidence shows a negative female-male gap in mental health, which partly derives from increasing risky addictive behaviours among girls and women (Hailemariam et al., 2021). The effect of substance use on mental health seems to derive from a complex combination of factors; social support, neighbourhood trust and dietary choice are some of the channels through which engaging in risky addictive behaviours influence mental health. Hence, policies aimed at promoting social capital and an overall healthy lifestyle could help in attenuating the negative effect of risky addictive behaviours on health.

Our study relies on a robust methodology, using representative samples of 11-, 13- and 15-year-old adolescents from 45 countries and regions. However, several limitations to our study are worth mentioning. First, our data are cross-sectional, our findings are correlational, and therefore no causality in the identified associations can be inferred. Finally, except for Canada, the sample included in our study was limited almost entirely to countries from the European region. Although we have a good variation in the countries included in our study, we acknowledge that there are cultures, especially throughout the Global South (Dados & Connell, 2012), where gender inequality is higher than in the countries included in the current sample. This means that opportunities for adolescent girls look much different than they do for boys, and there is a lack of important knowledge regarding gender differences in externalizing behaviours, such as substance use. Future studies should strive to address these gaps. Second, data were self-reported and therefore subject to biased estimates of substance use. However, adolescents seem to be accurate in their self-reported substance use (e.g., Harrison et al., 2007). The same report outlines that gender differences in under- or overreporting in substance use might emerge in late adolescence (18–25-years old) when young women are more likely to underreport (Harrison et al., 2007). Third, the results may be considered representative only for 11-13-15 year-old students attending regular schools and therefore not generalisable to adolescents not in school.

In summary, our study highlights that structural gender inequality is associated with gender differences in adolescent involvement in substance use. Considering the current large variations in the direction and magnitude of gender differences in adolescent substance use showed by our findings, when orienting the transition towards a more gender-equal society it is important to implement prevention programs focused on specific health behaviours and contexts. International health institutions and national health systems should adapt prevention policies to the specificity of local contexts, by using empirical evidence to inform programs and evaluating their effectiveness on the target outcomes (Hay et al., 2019).

## Funding

AC was supported by the European Regional Development Fund-Project “Effective Use of Social Research Studies for Practice” (No. CZ.02.1.01/0.0/0.0/16_025/0007294). FJE is supported by the Canada Research Chairs program. JI is supported by the 10.13039/501100000265Medical Research Council (MC_uu_00022/3) and the Chief Scientist Office of the Scottish Government Health and Social Care Directorate (SPHSU18).

## Author statement

Conceptualization (AC; FE, AV); Data curation (FE); Formal analysis (FE); Funding acquisition (AV); Methodology (AC,FE); Supervision (JI, AV); Roles/Writing - original draft (AC, FE, MdL, NC, ML, JI, AV); Writing - review & editing (AC, FE, MdL, NC, ML, JI, AV).

## Declaration of competing interest

The Authors declare that there is no conflict of interest.

## Data Availability

Data will be made available on request.
