# Supplementary material for: Structural gender inequality and gender differences in adolescent substance use: A multilevel study from 45 countries
Source: SSM Popul Health. 2022 Sep 6;19:101208. doi: 10.1016/j.ssmph.2022.101208 (PMC9482136; doi:10.1016/j.ssmph.2022.101208)
Supplement: Multimedia component 1 [file mmc1.docx]

**Supplementary tables and figures**

**Table S1**

**2018 Gender Inequality Index by Countries included in the study^^[[1]](#footnote-1)^^**

| **Country** | Value | Rank |
| --- | --- | --- |
|  | 2018 | 2018 |
| Switzerland | 0.037 | 1 |
| Sweden | 0.040 | 2 |
| Denmark | 0.040 | 2 |
| Netherlands | 0.041 | 4 |
| Norway | 0.044 | 5 |
| Belgium | 0.045 | 6 |
| Finland | 0.050 | 7 |
| France | 0.051 | 8 |
| Iceland | 0.057 | 9 |
| Slovenia | 0.069 | 12 |
| Italy | 0.069 | 12 |
| Austria | 0.073 | 14 |
| Spain | 0.074 | 15 |
| Luxembourg | 0.078 | 16 |
| Portugal | 0.081 | 17 |
| Canada | 0.083 | 18 |
| Germany | 0.084 | 19 |
| Cyprus | 0.086 | 20 |
| Estonia | 0.091 | 21 |
| Ireland | 0.093 | 22 |
| Israel | 0.100 | 24 |
| United Kingdom | 0.119 | 27 |
| Poland | 0.120 | 30 |
| Greece | 0.122 | 31 |
| Croatia | 0.122 | 31 |
| Lithuania | 0.124 | 33 |
| Czechia | 0.137 | 35 |
| North Macedonia | 0.145 | 36 |
| Latvia | 0.169 | 40 |
| Slovakia | 0.190 | 43 |
| Malta | 0.195 | 44 |
| Kazakhstan | 0.203 | 46 |
| Bulgaria | 0.218 | 48 |
| Moldova (Republic of) | 0.228 | 50 |
| Albania | 0.234 | 51 |
| Russian Federation | 0.255 | 54 |
| Hungary | 0.258 | 56 |
| Armenia | 0.259 | 57 |
| Ukraine | 0.284 | 60 |
| Turkey | 0.305 | 66 |
| Romania | 0.316 | 69 |
| Azerbaijan | 0.321 | 70 |
| Georgia | 0.351 | 75 |

**Table S2**

*Multilevel regression of substance use in adolescents including interactions of gender and gender inequality (Azerbaijan cases removed, HBSC 2017/18).*

|  | Smoking  (lifetime) | | Smoking  (past 30 days) | | | Alcohol use  (lifetime) | | Alcohol use  (past 30 days) | | Drunkenness  (lifetime) | | Drunkenness  (past 30 days) | |
| --- | --- | --- | --- | --- | --- | --- | --- | --- | --- | --- | --- | --- | --- |
|  | OR | 95% CI | OR | 95% CI | | OR | 95% CI | OR | 95% CI | OR | 95% CI | OR | 95% CI |
| Gender (male) | 1.04 | (0.98-1.10) | 0.91^*^ | | (0.84-0.98) | 1.28^***^ | (1.22-1.33) | 1.05 | (1.00-1.10) | 1.04 | (0.98-1.10) | 1.03 | (0.96-1.12) |
| Age group |  |  |  | |  |  |  |  |  |  |  |  |  |
| 11y | 1.00 | (1.00-1.00) | 1.00 | | (1.00-1.00) | 1.00 | (1.00-1.00) | 1.00 | (1.00-1.00) | 1.00 | (1.00-1.00) | 1.00 | (1.00-1.00) |
| 13y | 3.72^***^ | (3.54-3.92) | 3.33^***^ | | (3.08-3.60) | 3.13^***^ | (3.03-3.24) | 3.25^***^ | (3.10-3.41) | 2.94^***^ | (2.79-3.09) | 2.66^***^ | (2.45-2.88) |
| 15y | 12.32^***^ | (11.70-12.96) | 12.76^***^ | | (11.85-13.73) | 11.41^***^ | (11.01-11.82) | 12.72^***^ | (12.14-13.32) | 13.40^***^ | (12.75-14.08) | 12.08^***^ | (11.20-13.03) |
| Gender inequality (GII) | 0.19 | (0.01-4.43) | 0.41 | | (0.03-5.06) | 0.17 | (0.00-6.59) | 0.19 | (0.01-6.51) | 2.47 | (0.13-48.56) | 0.56 | (0.04-7.58) |
| Deprivation | 0.97 | (0.92-1.02) | 1.03 | | (0.97-1.11) | 0.65^***^ | (0.62-0.67) | 0.60^***^ | (0.57-0.63) | 0.71^***^ | (0.68-0.75) | 0.68^***^ | (0.63-0.73) |
| Country wealth (GDP pc) | 0.99 | (0.98-1.00) | 0.99 | | (0.99-1.00) | 0.99 | (0.98-1.01) | 0.99 | (0.98-1.00) | 0.99 | (0.98-1.00) | 0.99 | (0.98-1.00) |
| Gender * GII | 4.30^***^ | (2.87-6.45) | 5.94^***^ | | (3.50-10.06) | 1.70^***^ | (1.26-2.30) | 4.39^***^ | (3.08-6.24) | 5.62^***^ | (3.88-8.13) | 8.55^***^ | (5.04-14.52) |
|  |  |  |  | |  |  |  |  |  |  |  |  |  |
| Constant | 0.04^***^ | (0.02-0.09) | 0.02^***^ | | (0.01-0.03) | 0.22^**^ | (0.09-0.57) | 0.08^***^ | (0.03-0.20) | 0.04^***^ | (0.02-0.09) | 0.02^***^ | (0.01-0.03) |
|  |  |  |  |  | |  |  |  |  |  |  |  |  |
| Variances: |  |  |  |  | |  |  |  |  |  |  |  |  |
| Country | 0.33 | | 0.20 | | | 0.41 | | 0.42 | | 0.29 | | 0.22 | |
| School | 0.29 | | 0.37 | | | 0.55 | | 0.35 | | 0.31 | | 0.40 | |
| ICC (country) | 0.03 | | 0.02 | | | 0.05 | | 0.03 | | 0.03 | | 0.01 | |
| ICC (school) | 0.13 | | 0.09 | | | 0.20 | | 0.16 | | 0.14 | | 0.08 | |
| *n* | 209,973 | | 210,449 | | | 210,605 | | 210,972 | | 210,338 | | 209,931 | |

Note: OR = odds ratio. CI = confidence interval. ICC = intraclass correlations

**Table S3**

*Fixed effects regression of substance use in adolescents including interactions of gender and gender inequality (HBSC 2017/18).*

|  | Smoking  (lifetime) | | Smoking  (past 30 days) | | Alcohol use  (lifetime) | | Alcohol use  (past 30 days) | | Drunkenness  (lifetime) | | Drunkenness  (past 30 days) | |
| --- | --- | --- | --- | --- | --- | --- | --- | --- | --- | --- | --- | --- |
|  | OR | 95% CI | OR | 95% CI | OR | 95% CI | OR | 95% CI | OR | 95% CI | OR | 95% CI |
| Gender (male) | 0.99 | (0.93-1.06) | 0.86^***^ | (0.79-0.94) | 1.21^***^ | (1.16-1.27) | 0.99 | (0.94-1.05) | 1.00 | (0.94-1.06) | 0.96 | (0.88-1.04) |
| Age group |  |  |  |  |  |  |  |  |  |  |  |  |
| 11y | 1.00 |  | 1.00 |  | 1.00 |  | 1.00 |  | 1.00 |  | 1.00 |  |
| 13y | 3.70^***^ | (3.48-3.93) | 3.33^***^ | (3.05-3.64) | 3.00^***^ | (2.89-3.13) | 3.33^***^ | (3.16-3.51) | 3.05^***^ | (2.88-3.24) | 2.86^***^ | (2.62-3.13) |
| 15y | 11.75^***^ | (11.09-12.44) | 12.31^***^ | (11.34-13.37) | 10.30^***^ | (9.88-10.73) | 12.51^***^ | (11.89-13.16) | 13.34^***^ | (12.62-14.09) | 12.72^***^ | (11.71-13.82) |
| Deprivation | 1.06^*^ | (1.00-1.12) | 1.15^***^ | (1.07-1.23) | 0.64^***^ | (0.62-0.67) | 0.61^***^ | (0.59-0.64) | 0.76^***^ | (0.72-0.80) | 0.73^***^ | (0.68-0.78) |
| Gender*GII | 4.49^***^ | (2.87-7.02) | 6.35^***^ | (3.52-11.44) | 2.02^***^ | (1.43-2.84) | 5.01^***^ | (3.38-7.42) | 6.16^***^ | (4.05-9.37) | 9.22^***^ | (5.20-16.33) |
|  |  |  |  |  |  |  |  |  |  |  |  |  |
| Constant | 0.03^***^ | (0.02-0.03) | 0.01^***^ | (0.01-0.01) | 0.10^***^ | (0.08-0.11) | 0.04^***^ | (0.03-0.05) | 0.05^***^ | (0.04-0.06) | 0.01^***^ | (0.01-0.02) |
|  |  |  |  |  |  |  |  |  |  |  |  |  |
| *n* | 212469 | | 212938 | | 213053 | | 213445 | | 213445 | | 213445 | |

Note: OR = odds ratio. CI = confidence interval. Results are weighted and adjusted for school-level clustering and unmeasured country differences (dummy-coded).

**Table S4**

***Gender gap in prevalence of smoking (lifetime).***

|  | Boys | | | Girls | | | Gender |
| --- | --- | --- | --- | --- | --- | --- | --- |
| Country | n | % | 95% CI | n | % | 95% CI | Difference |
| Italy | 359 | 18.16 | [15.88,20.69] | 474 | 22.33 | [19.05,25.99] | -4.17 |
| Bulgaria | 446 | 21.09 | [19.06,23.27] | 554 | 24.68 | [22.20,27.33] | -3.59 |
| Hungary | 207 | 17.60 | [14.87,20.71] | 306 | 20.44 | [17.24,24.07] | -2.84 |
| Wales | 722 | 9.72 | [8.45,11.15] | 882 | 12.18 | [10.42,14.20] | -2.46 |
| Spain | 295 | 14.30 | [12.60,16.19] | 357 | 16.16 | [14.26,18.26] | -1.86 |
| Serbia | 254 | 13.57 | [11.50,15.94] | 298 | 15.32 | [12.61,18.50] | -1.75 |
| Czechia | 1121 | 19.45 | [17.83,21.18] | 1167 | 20.77 | [18.92,22.75] | -1.32 |
| Sweden | 217 | 11.02 | [9.28,13.05] | 250 | 12.34 | [10.39,14.60] | -1.32 |
| Greece | 282 | 14.87 | [12.69,17.34] | 309 | 16.12 | [13.63,18.96] | -1.25 |
| Canada | 550 | 7.77 | [6.57,9.17] | 580 | 8.37 | [7.02,9.96] | -0.60 |
| Scotland | 209 | 8.79 | [7.11,10.81] | 233 | 9.06 | [7.23,11.29] | -0.27 |
| Croatia | 551 | 21.87 | [19.36,24.59] | 537 | 22.14 | [19.22,25.37] | -0.27 |
| Germany | 295 | 14.69 | [12.58,17.09] | 337 | 14.83 | [12.62,17.35] | -0.14 |
| Austria | 286 | 14.28 | [12.01,16.89] | 290 | 14.15 | [11.93,16.72] | 0.13 |
| Portugal | 323 | 11.45 | [10.02,13.06] | 347 | 11.28 | [9.78,12.98] | 0.17 |
| Poland | 556 | 21.85 | [19.70,24.16] | 567 | 21.51 | [19.17,24.05] | 0.34 |
| Ireland | 153 | 8.01 | [6.46,9.89] | 140 | 7.49 | [5.96,9.38] | 0.52 |
| Luxembourg | 312 | 15.69 | [13.79,17.79] | 297 | 14.93 | [13.07,17.01] | 0.76 |
| Malta | 93 | 7.62 | [5.68,10.16] | 90 | 6.80 | [5.09,9.03] | 0.82 |
| Romania | 404 | 18.76 | [16.53,21.21] | 404 | 17.82 | [15.48,20.43] | 0.94 |
| Iceland | 219 | 6.48 | [4.74,8.80] | 188 | 5.50 | [3.86,7.77] | 0.98 |
| Belgium (VLG) | 184 | 8.95 | [7.47,10.68] | 158 | 7.43 | [6.08,9.06] | 1.52 |
| Belgium (WAL) | 314 | 11.86 | [10.39,13.50] | 273 | 10.09 | [8.63,11.76] | 1.77 |
| England | 175 | 10.63 | [8.61,13.06] | 137 | 8.78 | [6.93,11.06] | 1.85 |
| Latvia | 552 | 25.76 | [23.06,28.66] | 524 | 23.83 | [20.96,26.96] | 1.93 |
| Netherlands | 253 | 11.16 | [9.44,13.14] | 220 | 9.05 | [7.43,10.99] | 2.11 |
| Slovenia | 375 | 13.44 | [11.66,15.44] | 302 | 11.23 | [9.29,13.51] | 2.21 |
| Kazakhstan | 138 | 5.88 | [4.76,7.24] | 84 | 3.64 | [2.78,4.75] | 2.24 |
| France | 736 | 16.81 | [15.14,18.62] | 664 | 14.22 | [12.49,16.15] | 2.59 |
| Norway | 89 | 13.15 | [9.98,17.12] | 73 | 10.27 | [7.71,13.54] | 2.88 |
| Slovakia | 516 | 22.46 | [20.26,24.84] | 423 | 19.12 | [17.00,21.44] | 3.34 |
| Azerbaijan | 83 | 7.16 | [5.62,9.06] | 51 | 3.82 | [2.87,5.06] | 3.34 |
| Switzerland | 656 | 17.49 | [15.85,19.26] | 514 | 13.89 | [12.43,15.49] | 3.60 |
| Denmark | 177 | 11.74 | [9.55,14.34] | 128 | 7.90 | [6.09,10.19] | 3.84 |
| Russia | 272 | 14.90 | [12.90,17.14] | 215 | 10.78 | [9.28,12.48] | 4.12 |
| Finland | 319 | 20.13 | [17.18,23.45] | 248 | 15.50 | [12.71,18.77] | 4.63 |
| Lithuania | 693 | 37.08 | [32.52,41.88] | 593 | 32.04 | [26.87,37.69] | 5.04 |
| Macedonia | 324 | 14.57 | [12.23,17.28] | 222 | 9.45 | [7.78,11.44] | 5.12 |
| Estonia | 630 | 27.19 | [24.34,30.24] | 487 | 21.05 | [18.63,23.68] | 6.14 |
| Georgia | 231 | 11.29 | [9.34,13.59] | 103 | 4.94 | [3.71,6.55] | 6.35 |
| Ukraine | 606 | 18.74 | [17.03,20.59] | 388 | 11.67 | [10.27,13.24] | 7.07 |
| Israel | 336 | 14.55 | [12.45,16.94] | 180 | 5.68 | [4.71,6.84] | 8.87 |
| Moldova | 353 | 15.54 | [13.52,17.81] | 119 | 5.18 | [4.16,6.43] | 10.36 |
| Albania | 148 | 19.25 | [16.24,22.66] | 81 | 8.73 | [6.83,11.09] | 10.52 |
| Armenia | 284 | 13.23 | [11.46,15.23] | 51 | 2.24 | [1.64,3.04] | 10.99 |

**Table S5**

***Gender gap in prevalence of smoking (past 30 days).***

|  | Boys | | | Girls | | | Gender |
| --- | --- | --- | --- | --- | --- | --- | --- |
| Country | n | % | 95% CI | n | % | 95% CI | Difference |
| Bulgaria | 331 | 15.05 | [13.12,17.19] | 459 | 19.55 | [17.12,22.23] | -4.50 |
| Italy | 183 | 9.26 | [7.70,11.11] | 288 | 13.57 | [11.11,16.48] | -4.31 |
| Hungary | 96 | 8.14 | [6.48,10.18] | 165 | 11.04 | [8.83,13.72] | -2.90 |
| Czechia | 427 | 7.41 | [6.49,8.44] | 515 | 9.27 | [8.16,10.52] | -1.86 |
| Wales | 406 | 5.43 | [4.62,6.38] | 512 | 7.11 | [5.99,8.43] | -1.68 |
| Spain | 133 | 6.49 | [5.32,7.89] | 172 | 7.82 | [6.55,9.32] | -1.33 |
| Austria | 122 | 6.14 | [4.83,7.78] | 152 | 7.41 | [5.90,9.26] | -1.27 |
| Poland | 214 | 8.41 | [7.13,9.89] | 250 | 9.50 | [8.07,11.16] | -1.09 |
| England | 73 | 4.28 | [3.19,5.73] | 84 | 5.33 | [4.10,6.90] | -1.05 |
| Germany | 130 | 6.47 | [5.22,7.99] | 170 | 7.47 | [6.12,9.09] | -1.00 |
| Serbia | 172 | 9.08 | [7.36,11.15] | 194 | 9.92 | [7.72,12.67] | -0.84 |
| Greece | 141 | 7.47 | [6.01,9.25] | 159 | 8.27 | [6.64,10.27] | -0.80 |
| Sweden | 113 | 5.71 | [4.47,7.26] | 131 | 6.46 | [5.18,8.04] | -0.75 |
| Canada | 273 | 3.60 | [2.87,4.49] | 313 | 4.35 | [3.58,5.28] | -0.75 |
| France | 289 | 6.53 | [5.37,7.93] | 306 | 6.97 | [5.85,8.28] | -0.44 |
| Latvia | 213 | 9.98 | [8.35,11.88] | 228 | 10.40 | [8.64,12.46] | -0.42 |
| Scotland | 100 | 4.42 | [3.32,5.86] | 114 | 4.61 | [3.39,6.24] | -0.19 |
| Belgium (WAL) | 142 | 5.36 | [4.52,6.35] | 148 | 5.47 | [4.52,6.62] | -0.11 |
| Luxembourg | 161 | 8.09 | [6.63,9.84] | 162 | 8.13 | [6.76,9.75] | -0.04 |
| Ireland | 74 | 3.88 | [2.95,5.08] | 68 | 3.65 | [2.74,4.83] | 0.23 |
| Estonia | 211 | 9.18 | [7.72,10.87] | 207 | 8.95 | [7.56,10.55] | 0.23 |
| Romania | 243 | 11.22 | [9.50,13.20] | 247 | 10.81 | [9.15,12.73] | 0.41 |
| Slovakia | 233 | 10.16 | [8.71,11.81] | 215 | 9.73 | [8.23,11.47] | 0.43 |
| Croatia | 314 | 12.43 | [10.48,14.67] | 291 | 11.94 | [9.83,14.43] | 0.49 |
| Malta | 53 | 4.33 | [2.96,6.29] | 50 | 3.77 | [2.51,5.61] | 0.56 |
| Slovenia | 187 | 6.68 | [5.38,8.25] | 165 | 6.11 | [4.77,7.79] | 0.57 |
| Portugal | 156 | 5.53 | [4.60,6.63] | 143 | 4.65 | [3.85,5.61] | 0.88 |
| Finland | 146 | 8.91 | [7.27,10.87] | 128 | 8.01 | [6.26,10.19] | 0.90 |
| Belgium (VLG) | 97 | 4.70 | [3.67,6.00] | 80 | 3.73 | [2.89,4.80] | 0.97 |
| Netherlands | 131 | 5.77 | [4.57,7.25] | 114 | 4.66 | [3.64,5.94] | 1.11 |
| Azerbaijan | 42 | 3.64 | [2.62,5.03] | 33 | 2.47 | [1.73,3.53] | 1.17 |
| Switzerland | 260 | 6.92 | [5.93,8.06] | 213 | 5.75 | [4.83,6.83] | 1.17 |
| Kazakhstan | 88 | 3.73 | [2.94,4.74] | 56 | 2.41 | [1.86,3.12] | 1.32 |
| Lithuania | 284 | 15.34 | [12.66,18.47] | 259 | 13.92 | [11.07,17.37] | 1.42 |
| Iceland | 126 | 3.71 | [2.72,5.04] | 77 | 2.25 | [1.46,3.47] | 1.46 |
| Denmark | 93 | 6.14 | [4.75,7.91] | 75 | 4.62 | [3.41,6.22] | 1.52 |
| Norway | 44 | 6.47 | [4.49,9.23] | 34 | 4.78 | [3.28,6.92] | 1.69 |
| Russia | 138 | 7.51 | [6.11,9.20] | 102 | 5.08 | [4.21,6.11] | 2.43 |
| Macedonia | 175 | 7.94 | [6.43,9.76] | 118 | 5.02 | [3.92,6.41] | 2.92 |
| Georgia | 148 | 7.14 | [5.55,9.15] | 69 | 3.27 | [2.40,4.44] | 3.87 |
| Ukraine | 288 | 8.89 | [7.75,10.18] | 162 | 4.87 | [4.04,5.86] | 4.02 |
| Moldova | 157 | 6.91 | [5.72,8.32] | 49 | 2.13 | [1.47,3.06] | 4.78 |
| Albania | 80 | 10.36 | [8.21,13.01] | 35 | 3.75 | [2.71,5.17] | 6.61 |
| Israel | 227 | 9.97 | [8.26,11.98] | 104 | 3.26 | [2.54,4.18] | 6.71 |
| Armenia | 183 | 8.43 | [6.94,10.19] | 20 | 0.88 | [0.56,1.36] | 7.55 |

**Table S6**

***Gender gap in prevalence of alcohol use (lifetime).***

|  | Boys | | | Girls | | | Gender |
| --- | --- | --- | --- | --- | --- | --- | --- |
| Country | n | % | 95% CI | n | % | 95% CI | Difference |
| Lithuania | 799 | 42.55 | [37.97,47.26] | 819 | 44.27 | [37.90,50.84] | -1.72 |
| Canada | 1850 | 35.52 | [32.63,38.51] | 2081 | 36.59 | [33.51,39.79] | -1.07 |
| Finland | 475 | 46.42 | [41.65,51.24] | 490 | 47.47 | [42.32,52.66] | -1.05 |
| Ukraine | 1056 | 32.85 | [30.64,35.14] | 1121 | 33.90 | [31.54,36.33] | -1.05 |
| Poland | 871 | 34.10 | [31.15,37.19] | 923 | 35.00 | [31.64,38.52] | -0.90 |
| Kazakhstan | 106 | 4.44 | [3.47,5.68] | 102 | 4.35 | [3.32,5.67] | 0.09 |
| Latvia | 892 | 41.43 | [38.17,44.76] | 903 | 41.12 | [37.44,44.90] | 0.31 |
| Sweden | 520 | 26.54 | [23.69,29.61] | 520 | 25.74 | [22.63,29.12] | 0.80 |
| Russia | 332 | 18.38 | [16.11,20.89] | 347 | 17.50 | [15.40,19.81] | 0.88 |
| Germany | 822 | 41.10 | [37.29,45.02] | 897 | 39.66 | [35.59,43.87] | 1.44 |
| Spain | 798 | 38.95 | [36.16,41.80] | 810 | 36.99 | [33.97,40.11] | 1.96 |
| Bulgaria | 936 | 47.51 | [44.29,50.76] | 967 | 45.25 | [41.63,48.92] | 2.26 |
| Belgium (VLG) | 762 | 37.15 | [34.11,40.30] | 735 | 34.60 | [31.12,38.27] | 2.55 |
| England | 715 | 42.97 | [38.53,47.52] | 598 | 40.35 | [35.58,45.30] | 2.62 |
| Ireland | 460 | 24.11 | [21.07,27.44] | 397 | 21.36 | [18.12,24.99] | 2.75 |
| Iceland | 592 | 17.59 | [14.94,20.59] | 497 | 14.63 | [10.81,19.50] | 2.96 |
| Scotland | 936 | 40.58 | [36.94,44.33] | 925 | 37.45 | [33.34,41.76] | 3.13 |
| Portugal | 962 | 34.51 | [31.89,37.22] | 940 | 30.99 | [28.18,33.95] | 3.52 |
| Wales | 3754 | 50.74 | [47.21,54.25] | 3425 | 47.19 | [42.73,51.69] | 3.55 |
| Czechia | 2676 | 46.85 | [44.52,49.20] | 2405 | 43.10 | [40.43,45.82] | 3.75 |
| Malta | 504 | 41.69 | [36.65,46.90] | 496 | 37.75 | [32.01,43.85] | 3.94 |
| Norway | 197 | 29.32 | [24.81,34.27] | 178 | 25.36 | [21.18,30.04] | 3.96 |
| Estonia | 954 | 41.70 | [38.35,45.12] | 868 | 37.72 | [34.44,41.12] | 3.98 |
| Luxembourg | 669 | 33.87 | [31.16,36.69] | 597 | 29.88 | [26.91,33.03] | 3.99 |
| Austria | 787 | 39.27 | [35.29,43.40] | 720 | 34.97 | [30.63,39.57] | 4.30 |
| Slovakia | 821 | 36.01 | [33.31,38.80] | 685 | 31.24 | [28.41,34.21] | 4.77 |
| Croatia | 1174 | 47.21 | [43.79,50.65] | 1003 | 41.65 | [37.81,45.60] | 5.56 |
| Hungary | 888 | 51.54 | [47.34,55.71] | 894 | 45.78 | [40.97,50.66] | 5.76 |
| Italy | 839 | 42.81 | [39.29,46.40] | 783 | 36.88 | [32.98,40.96] | 5.93 |
| Greece | 1050 | 55.73 | [51.76,59.64] | 945 | 49.58 | [45.05,54.11] | 6.15 |
| Belgium (WAL) | 1020 | 38.99 | [36.15,41.91] | 862 | 32.28 | [29.59,35.10] | 6.71 |
| Azerbaijan | 117 | 10.34 | [8.27,12.87] | 43 | 3.26 | [2.34,4.54] | 7.08 |
| Netherlands | 817 | 35.79 | [32.47,39.24] | 691 | 28.40 | [25.11,31.95] | 7.39 |
| Switzerland | 1576 | 42.38 | [39.89,44.91] | 1272 | 34.63 | [32.16,37.19] | 7.75 |
| Slovenia | 1248 | 44.35 | [40.99,47.76] | 984 | 36.35 | [32.67,40.21] | 8.00 |
| France | 2359 | 53.01 | [50.56,55.45] | 2020 | 44.21 | [41.55,46.92] | 8.80 |
| Denmark | 621 | 41.48 | [37.06,46.05] | 524 | 32.47 | [27.87,37.43] | 9.01 |
| Macedonia | 653 | 29.86 | [26.09,33.92] | 476 | 20.52 | [17.20,24.29] | 9.34 |
| Serbia | 924 | 50.38 | [46.61,54.15] | 784 | 40.66 | [36.26,45.23] | 9.72 |
| Romania | 977 | 46.13 | [42.74,49.56] | 811 | 35.90 | [32.71,39.22] | 10.23 |
| Moldova | 840 | 37.82 | [34.46,41.30] | 579 | 25.71 | [22.81,28.85] | 12.11 |
| Georgia | 848 | 42.57 | [39.03,46.19] | 622 | 30.33 | [27.02,33.85] | 12.24 |
| Albania | 264 | 34.55 | [30.64,38.70] | 200 | 21.88 | [18.40,25.82] | 12.67 |
| Armenia | 855 | 40.79 | [37.75,43.90] | 540 | 24.19 | [21.41,27.22] | 16.60 |
| Israel | 1102 | 31.83 | [29.41,34.35] | 649 | 14.44 | [12.86,16.19] | 17.39 |

**Table S7**

***Gender gap in prevalence of alcohol use (past 30 days).***

|  | Boys | | | Girls | | | Gender |
| --- | --- | --- | --- | --- | --- | --- | --- |
| Country | n | % | 95% CI | n | % | 95% CI | Difference |
| Latvia | 388 | 18.03 | [15.80,20.49] | 504 | 22.96 | [20.12,26.07] | -4.93 |
| Canada | 840 | 16.47 | [14.31,18.88] | 1045 | 19.11 | [16.78,21.66] | -2.64 |
| Scotland | 398 | 17.26 | [14.67,20.21] | 468 | 19.44 | [16.36,22.94] | -2.18 |
| Germany | 483 | 24.16 | [20.90,27.76] | 589 | 25.98 | [22.60,29.67] | -1.82 |
| Ukraine | 524 | 16.37 | [14.78,18.11] | 590 | 18.15 | [16.37,20.07] | -1.78 |
| Sweden | 200 | 10.19 | [8.43,12.27] | 242 | 11.95 | [9.97,14.26] | -1.76 |
| Belgium (VLG) | 441 | 21.49 | [18.91,24.32] | 490 | 22.94 | [19.88,26.32] | -1.45 |
| Poland | 387 | 15.16 | [13.01,17.59] | 438 | 16.61 | [14.35,19.14] | -1.45 |
| Lithuania | 298 | 16.02 | [13.45,18.97] | 319 | 17.12 | [13.78,21.08] | -1.10 |
| Austria | 410 | 20.67 | [17.38,24.39] | 447 | 21.67 | [18.28,25.49] | -1.00 |
| Spain | 366 | 18.02 | [15.89,20.37] | 402 | 18.48 | [16.24,20.96] | -0.46 |
| England | 322 | 20.09 | [16.80,23.84] | 311 | 20.52 | [16.74,24.91] | -0.43 |
| Ireland | 165 | 8.77 | [7.10,10.79] | 170 | 9.18 | [7.47,11.25] | -0.41 |
| Wales | 1954 | 26.82 | [23.92,29.94] | 1922 | 26.77 | [23.28,30.58] | 0.05 |
| Kazakhstan | 56 | 2.34 | [1.73,3.16] | 45 | 1.92 | [1.33,2.76] | 0.42 |
| Iceland | 202 | 6.01 | [4.41,8.13] | 179 | 5.27 | [3.45,7.97] | 0.74 |
| Estonia | 366 | 16.05 | [14.08,18.22] | 353 | 15.30 | [13.37,17.45] | 0.75 |
| Portugal | 521 | 18.48 | [16.45,20.68] | 541 | 17.59 | [15.54,19.86] | 0.89 |
| Malta | 268 | 22.17 | [17.95,27.05] | 277 | 21.06 | [16.68,26.24] | 1.11 |
| Finland | 260 | 25.76 | [21.82,30.13] | 248 | 24.26 | [20.49,28.47] | 1.50 |
| Russia | 165 | 9.06 | [7.46,10.97] | 148 | 7.45 | [6.27,8.83] | 1.61 |
| Bulgaria | 716 | 32.55 | [29.91,35.29] | 726 | 30.92 | [27.90,34.11] | 1.63 |
| Netherlands | 394 | 17.37 | [14.82,20.25] | 385 | 15.70 | [13.33,18.40] | 1.67 |
| Czechia | 1503 | 25.78 | [23.92,27.72] | 1348 | 24.03 | [22.08,26.10] | 1.75 |
| Norway | 98 | 14.45 | [11.06,18.67] | 87 | 12.24 | [9.40,15.77] | 2.21 |
| Hungary | 461 | 26.69 | [23.54,30.10] | 476 | 24.39 | [20.80,28.36] | 2.30 |
| Luxembourg | 349 | 17.70 | [15.69,19.91] | 302 | 15.11 | [13.12,17.33] | 2.59 |
| Slovakia | 415 | 18.24 | [16.29,20.37] | 336 | 15.31 | [13.39,17.46] | 2.93 |
| Switzerland | 727 | 19.49 | [17.64,21.48] | 597 | 16.21 | [14.54,18.05] | 3.28 |
| Denmark | 356 | 23.58 | [19.80,27.82] | 327 | 20.16 | [16.45,24.47] | 3.42 |
| Italy | 507 | 25.78 | [22.81,28.98] | 464 | 21.90 | [18.90,25.22] | 3.88 |
| France | 1110 | 25.20 | [23.13,27.38] | 950 | 20.60 | [18.51,22.86] | 4.60 |
| Belgium (WAL) | 620 | 23.75 | [21.36,26.31] | 510 | 19.01 | [16.92,21.29] | 4.74 |
| Greece | 606 | 32.22 | [28.78,35.85] | 522 | 27.34 | [23.96,31.02] | 4.88 |
| Azerbaijan | 78 | 6.81 | [5.30,8.71] | 23 | 1.73 | [1.12,2.67] | 5.08 |
| Slovenia | 702 | 24.60 | [21.77,27.67] | 537 | 19.52 | [16.79,22.56] | 5.08 |
| Croatia | 700 | 28.01 | [25.23,30.97] | 543 | 22.41 | [19.56,25.55] | 5.60 |
| Serbia | 668 | 35.65 | [31.84,39.64] | 527 | 27.16 | [23.41,31.27] | 8.49 |
| Macedonia | 468 | 21.21 | [18.07,24.72] | 292 | 12.49 | [10.12,15.32] | 8.72 |
| Israel | 417 | 14.36 | [12.26,16.75] | 224 | 5.49 | [4.45,6.74] | 8.87 |
| Moldova | 485 | 21.57 | [19.26,24.07] | 285 | 12.51 | [10.78,14.48] | 9.06 |
| Romania | 645 | 30.25 | [27.34,33.34] | 463 | 20.32 | [17.92,22.94] | 9.93 |
| Albania | 160 | 20.65 | [17.86,23.74] | 96 | 10.39 | [8.17,13.13] | 10.26 |
| Georgia | 547 | 26.85 | [24.01,29.90] | 342 | 16.29 | [14.08,18.77] | 10.56 |
| Armenia | 591 | 27.75 | [25.02,30.64] | 290 | 12.87 | [10.90,15.13] | 14.88 |

**Table S8**

***Gender gap in prevalence of drunkenness (lifetime).***

|  | Boys | | | Girls | | | Gender |
| --- | --- | --- | --- | --- | --- | --- | --- |
| Country | n | % | 95% CI | n | % | 95% CI | Difference |
| Wales | 1013 | 47.16 | [44.52,49.82] | 1063 | 50.57 | [47.65,53.48] | -3.41 |
| Poland | 361 | 14.13 | [12.32,16.17] | 444 | 16.81 | [14.61,19.27] | -2.68 |
| Spain | 284 | 13.78 | [12.04,15.72] | 357 | 16.21 | [14.16,18.50] | -2.43 |
| Sweden | 177 | 9.00 | [7.39,10.91] | 208 | 10.30 | [8.46,12.48] | -1.30 |
| Canada | 885 | 16.45 | [14.32,18.84] | 1052 | 17.57 | [15.30,20.09] | -1.12 |
| Finland | 166 | 34.33 | [28.99,40.11] | 172 | 35.20 | [30.26,40.47] | -0.87 |
| Ireland | 191 | 10.01 | [8.03,12.41] | 200 | 10.71 | [8.65,13.19] | -0.70 |
| Germany | 352 | 17.56 | [14.87,20.61] | 410 | 18.04 | [15.30,21.14] | -0.48 |
| Latvia | 428 | 19.72 | [17.25,22.46] | 446 | 20.15 | [17.58,23.00] | -0.43 |
| Scotland | 406 | 17.73 | [14.90,20.96] | 442 | 17.89 | [14.94,21.28] | -0.16 |
| Wales | 1694 | 23.74 | [20.88,26.86] | 1675 | 23.70 | [20.42,27.32] | 0.04 |
| Lithuania | 462 | 24.52 | [20.77,28.71] | 455 | 24.41 | [19.60,29.95] | 0.11 |
| England | 288 | 17.45 | [14.24,21.19] | 271 | 17.32 | [14.43,20.64] | 0.13 |
| Portugal | 296 | 10.52 | [8.96,12.32] | 318 | 10.38 | [8.98,11.95] | 0.14 |
| Bulgaria | 602 | 28.38 | [25.81,31.10] | 636 | 27.99 | [25.20,30.96] | 0.39 |
| Kazakhstan | 78 | 3.29 | [2.54,4.24] | 66 | 2.81 | [2.08,3.79] | 0.48 |
| Italy | 287 | 14.49 | [12.42,16.84] | 297 | 14.00 | [11.70,16.66] | 0.49 |
| Iceland | 181 | 5.37 | [3.49,8.17] | 165 | 4.85 | [2.86,8.11] | 0.52 |
| Russia | 190 | 10.67 | [9.00,12.62] | 188 | 9.64 | [8.25,11.24] | 1.03 |
| Netherlands | 249 | 11.04 | [9.12,13.29] | 244 | 9.85 | [8.06,11.98] | 1.19 |
| Hungary | 366 | 21.13 | [18.13,24.48] | 391 | 19.85 | [16.74,23.38] | 1.28 |
| Luxembourg | 258 | 12.89 | [11.09,14.94] | 231 | 11.53 | [9.81,13.50] | 1.36 |
| Ukraine | 559 | 17.28 | [15.57,19.14] | 523 | 15.91 | [14.34,17.62] | 1.37 |
| Austria | 262 | 17.88 | [14.22,22.25] | 270 | 16.48 | [13.23,20.35] | 1.40 |
| Czechia | 1249 | 21.38 | [19.64,23.23] | 1107 | 19.59 | [17.86,21.45] | 1.79 |
| Norway | 95 | 14.01 | [10.52,18.42] | 86 | 12.06 | [9.17,15.70] | 1.95 |
| Belgium (WAL) | 389 | 15.14 | [13.51,16.92] | 343 | 12.89 | [11.28,14.70] | 2.25 |
| Estonia | 454 | 19.60 | [17.14,22.33] | 396 | 17.00 | [14.65,19.63] | 2.60 |
| Greece | 368 | 19.39 | [16.85,22.21] | 317 | 16.51 | [14.10,19.24] | 2.88 |
| France | 487 | 11.26 | [9.76,12.94] | 364 | 8.31 | [7.10,9.69] | 2.95 |
| Malta | 197 | 16.19 | [12.71,20.39] | 174 | 13.13 | [10.18,16.79] | 3.06 |
| Belgium (VLG) | 306 | 14.81 | [12.82,17.05] | 248 | 11.61 | [9.77,13.73] | 3.20 |
| Slovakia | 419 | 18.22 | [16.20,20.42] | 330 | 14.90 | [12.96,17.06] | 3.32 |
| Denmark | 270 | 18.02 | [14.47,22.23] | 232 | 14.41 | [11.37,18.09] | 3.61 |
| Azerbaijan | 82 | 7.16 | [5.65,9.02] | 37 | 2.79 | [1.97,3.95] | 4.37 |
| Switzerland | 527 | 14.06 | [12.48,15.80] | 337 | 9.11 | [7.94,10.43] | 4.95 |
| Macedonia | 302 | 13.64 | [11.40,16.24] | 197 | 8.41 | [6.63,10.61] | 5.23 |
| Slovenia | 617 | 21.82 | [19.21,24.69] | 430 | 15.82 | [13.35,18.66] | 6.00 |
| Croatia | 674 | 26.59 | [23.68,29.72] | 502 | 20.53 | [17.93,23.41] | 6.06 |
| Romania | 407 | 19.05 | [16.85,21.46] | 283 | 12.34 | [10.59,14.33] | 6.71 |
| Israel | 352 | 10.78 | [9.32,12.43] | 177 | 3.69 | [3.00,4.52] | 7.09 |
| Serbia | 502 | 26.60 | [23.29,30.21] | 358 | 18.32 | [15.44,21.60] | 8.28 |
| Albania | 190 | 24.64 | [21.60,27.96] | 141 | 15.06 | [12.45,18.11] | 9.58 |
| Moldova | 537 | 23.41 | [20.85,26.18] | 283 | 12.29 | [10.56,14.25] | 11.12 |
| Armenia | 640 | 29.92 | [27.36,32.61] | 358 | 15.74 | [13.70,18.03] | 14.18 |
| Georgia | 841 | 41.10 | [37.54,44.76] | 535 | 25.76 | [22.58,29.21] | 15.34 |

**Table S9**

***Gender gap in prevalence of drunkenness (past 30 days).***

|  | Boys | | | Girls | | | Gender |
| --- | --- | --- | --- | --- | --- | --- | --- |
| Country | n | % | 95% CI | n | % | 95% CI | Difference |
| Wales | 686 | 9.81 | [8.33,11.53] | 786 | 11.47 | [9.61,13.63] | -1.66 |
| Scotland | 155 | 6.83 | [5.33,8.70] | 200 | 8.45 | [6.46,10.97] | -1.62 |
| Canada | 393 | 6.64 | [5.40,8.14] | 484 | 7.99 | [6.74,9.45] | -1.35 |
| Latvia | 138 | 6.42 | [5.15,7.98] | 171 | 7.76 | [6.34,9.46] | -1.34 |
| England | 90 | 5.81 | [3.98,8.41] | 109 | 6.98 | [5.33,9.08] | -1.17 |
| Italy | 95 | 4.80 | [3.75,6.12] | 119 | 5.59 | [3.95,7.86] | -0.79 |
| Sweden | 75 | 3.82 | [2.83,5.13] | 91 | 4.50 | [3.49,5.80] | -0.68 |
| Spain | 98 | 4.81 | [3.86,5.99] | 119 | 5.45 | [4.41,6.72] | -0.64 |
| Poland | 144 | 5.65 | [4.62,6.90] | 156 | 5.93 | [4.81,7.29] | -0.28 |
| Austria | 105 | 7.32 | [5.35,9.94] | 114 | 7.02 | [5.23,9.38] | 0.30 |
| Ireland | 68 | 3.58 | [2.58,4.96] | 60 | 3.24 | [2.42,4.34] | 0.34 |
| Czechia | 471 | 7.99 | [7.02,9.09] | 422 | 7.48 | [6.58,8.50] | 0.51 |
| Luxembourg | 89 | 4.48 | [3.56,5.63] | 79 | 3.96 | [3.07,5.11] | 0.52 |
| Portugal | 136 | 4.82 | [3.86,6.01] | 131 | 4.26 | [3.49,5.19] | 0.56 |
| Germany | 173 | 8.63 | [6.92,10.71] | 182 | 8.04 | [6.52,9.88] | 0.59 |
| Netherlands | 121 | 5.37 | [4.22,6.82] | 115 | 4.67 | [3.62,5.99] | 0.70 |
| Iceland | 93 | 2.75 | [1.88,4.01] | 66 | 1.94 | [1.16,3.24] | 0.81 |
| Estonia | 159 | 6.96 | [5.80,8.31] | 139 | 6.03 | [4.89,7.40] | 0.93 |
| Ukraine | 204 | 6.32 | [5.38,7.40] | 173 | 5.37 | [4.50,6.40] | 0.95 |
| Kazakhstan | 56 | 2.35 | [1.72,3.21] | 30 | 1.28 | [0.84,1.93] | 1.07 |
| Hungary | 168 | 9.73 | [8.00,11.80] | 169 | 8.64 | [6.92,10.73] | 1.09 |
| France | 170 | 4.17 | [3.33,5.21] | 132 | 3.03 | [2.36,3.89] | 1.14 |
| Lithuania | 187 | 10.01 | [8.04,12.38] | 158 | 8.52 | [6.37,11.31] | 1.49 |
| Slovakia | 171 | 7.53 | [6.35,8.90] | 132 | 5.99 | [4.90,7.30] | 1.54 |
| Belgium (WAL) | 161 | 6.30 | [5.32,7.45] | 124 | 4.70 | [3.81,5.78] | 1.60 |
| Bulgaria | 379 | 17.23 | [15.22,19.44] | 364 | 15.50 | [13.62,17.60] | 1.73 |
| Russia | 82 | 4.59 | [3.60,5.83] | 54 | 2.77 | [2.07,3.69] | 1.82 |
| Malta | 73 | 6.02 | [4.42,8.15] | 54 | 4.08 | [2.73,6.07] | 1.94 |
| Slovenia | 263 | 9.27 | [7.65,11.18] | 198 | 7.31 | [5.84,9.12] | 1.96 |
| Belgium (VLG) | 150 | 7.24 | [5.99,8.72] | 113 | 5.27 | [4.15,6.67] | 1.97 |
| Denmark | 156 | 10.30 | [7.75,13.57] | 132 | 8.13 | [6.13,10.71] | 2.17 |
| Norway | 59 | 8.63 | [5.93,12.39] | 46 | 6.42 | [4.38,9.32] | 2.21 |
| Switzerland | 192 | 5.13 | [4.31,6.10] | 107 | 2.89 | [2.30,3.63] | 2.24 |
| Azerbaijan | 54 | 4.69 | [3.49,6.28] | 30 | 2.27 | [1.50,3.41] | 2.42 |
| Greece | 153 | 8.06 | [6.59,9.82] | 100 | 5.21 | [4.11,6.57] | 2.85 |
| Macedonia | 139 | 6.28 | [5.08,7.75] | 79 | 3.37 | [2.48,4.56] | 2.91 |
| Croatia | 267 | 10.62 | [9.07,12.40] | 174 | 7.14 | [5.73,8.86] | 3.48 |
| Moldova | 146 | 6.49 | [5.36,7.84] | 66 | 2.88 | [2.17,3.82] | 3.61 |
| Romania | 174 | 8.11 | [6.92,9.49] | 99 | 4.31 | [3.42,5.42] | 3.80 |
| Finland | 84 | 17.77 | [14.40,21.73] | 69 | 13.87 | [10.85,17.57] | 3.90 |
| Albania | 68 | 8.84 | [7.08,11.00] | 39 | 4.17 | [3.00,5.77] | 4.67 |
| Israel | 257 | 7.75 | [6.49,9.22] | 123 | 2.57 | [2.06,3.19] | 5.18 |
| Serbia | 291 | 15.35 | [13.07,17.94] | 181 | 9.24 | [7.42,11.46] | 6.11 |
| Georgia | 333 | 16.74 | [14.55,19.19] | 172 | 8.29 | [6.75,10.14] | 8.45 |
| Armenia | 258 | 12.19 | [10.47,14.15] | 65 | 2.91 | [2.26,3.73] | 9.28 |
| Armenia | 258 | 12.19 | [10.47,14.15] | 65 | 2.91 | [2.26,3.73] | 9.28 |

**Figure S1.** Odds ratios of smoking (lifetime and last 30 days) in adolescents in the Health Behaviour in School-aged Children study, 2017/18. Gender is coded 0 = girls, 1 = boys.

**Figure S2.** Odds ratios of alcohol use (lifetime and last 30 days) in adolescents in the Health Behaviour in School-aged Children study, 2017/18. Gender is coded 0 = girls, 1 = boys.

**Figure S3.** Odds ratios of drunkenness (lifetime and last 30 days) in adolescents in the Health Behaviour in School-aged Children study, 2017/18. Gender is coded 0 = girls, 1 = boys.

**Figure S4.** Country-level correlations in the prevalence of substance use in boys versus girls. Countries further off the diagonal line have larger gender differences (n = 45).

r = .29

r = .29

r = .24

r = .39**

r = .48**

r = .43*

**Figure S5.** Country-level correlations of gender differences in substance use ( prevalence for boys minus prevalence for girls) and country gender inequality (n = 45). *p < 0.05. **p < 0.01.

1. Data retrieved from: <http://data.un.org/DocumentData.aspx?id=415> [↑](#footnote-ref-1)
